# Evaluation of Diffusion-Tensor Imaging-Based Global Search and Tractography for Tumor Surgery Close to the Language System

**DOI:** 10.1371/journal.pone.0050132

**Published:** 2013-01-07

**Authors:** Mirco Richter, Amir Zolal, Oliver Ganslandt, Michael Buchfelder, Christopher Nimsky, Dorit Merhof

**Affiliations:** 1 Visual Computing, University of Konstanz, Konstanz, Germany; 2 Department of Neurosurgery, University of J. E. Purkyn and Masaryk Hospital, Ústí, Czech Republic; 3 Department of Neurosurgery, University of Erlangen-Nuremberg, Erlangen, Germany; 4 Department of Neurosurgery, University of Marburg, Marburg/Lahn, Germany; Centre Hospitalier Universitaire Vaudois Lausanne - CHUV, UNIL, Switzerland

## Abstract

Pre-operative planning and intra-operative guidance in neurosurgery require detailed information about the location of functional areas and their anatomo-functional connectivity. In particular, regarding the language system, post-operative deficits such as aphasia can be avoided. By combining functional magnetic resonance imaging and diffusion tensor imaging, the connectivity between functional areas can be reconstructed by tractography techniques that need to cope with limitations such as limited resolution and low anisotropic diffusion close to functional areas. Tumors pose particular challenges because of edema, displacement effects on brain tissue and infiltration of white matter. Under these conditions, standard fiber tracking methods reconstruct pathways of insufficient quality. Therefore, robust global or probabilistic approaches are required. In this study, two commonly used standard fiber tracking algorithms, streamline propagation and tensor deflection, were compared with a previously published global search, Gibbs tracking and a connection-oriented probabilistic tractography approach. All methods were applied to reconstruct neuronal pathways of the language system of patients undergoing brain tumor surgery, and control subjects. Connections between Broca and Wernicke areas via the arcuate fasciculus (AF) and the inferior fronto-occipital fasciculus (IFOF) were validated by a clinical expert to ensure anatomical feasibility, and compared using distance- and diffusion-based similarity metrics to evaluate their agreement on pathway locations. For both patients and controls, a strong agreement between all methods was observed regarding the location of the AF. In case of the IFOF however, standard fiber tracking and Gibbs tracking predominantly identified the inferior longitudinal fasciculus that plays a secondary role in semantic language processing. In contrast, global search resolved connections in almost every case via the IFOF which could be confirmed by probabilistic fiber tracking. The results show that regarding the language system, our global search is superior to clinically applied conventional fiber tracking strategies with results similar to time-consuming global or probabilistic approaches.

## Introduction

The neurological network of the human brain comprises complex functional units such as the language system, which consists of multiple functional areas connected via white matter pathways. These subcortical networks are of major interest for pre-operative neurosurgical planning and intra-operative guidance close to eloquent structures to prevent neurological deficits such as post-operative aphasia.

Insight into the general architecture of the language system is provided by various anatomo-functional connectivity studies with invasive and non-invasive techniques. Intra-operative subcortical mapping (ISM) locates pathways by inducing temporal neurological dysfunctions with electrical stimulation [1]. In contrast, with image data from functional magnetic resonance imaging (fMRI), functional nodes and their causal associations can be identified by various types of correlation analyses [2]–[4].

Both types of functional connectivity studies have been supplemented by diffusion tensor imaging (DTI) [3], [5], which is a magnetic resonance imaging (MRI) technique that measures the diffusion characteristics of water molecules [6]. In brain parenchyma, anisotropic diffusion is observed in aligned microstructures such as white matter pathways, whereas isotropic diffusion is dominant in gray matter and other tissues. To characterize these differences, DTI encodes information about the direction and intensity of diffusion by a 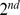-order tensor 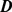. Based on 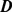, the trajectories of neuronal pathways can be reconstructed by fiber tractography.

For the detection of language related white matter tracts, ISM can be applied during awake surgery. In such a procedure, the patient undergoes repetitive language testing while the subcortical surface of the resection cavity is being stimulated. This invasive method can also be used to verify the results of DTI fiber tractography [1], [5]. In contrast, with the combination of fMRI-based statistical functional mapping and DTI-based neuronal fiber reconstruction, functional and anatomical connectivity can be resolved without additional invasive steps. Recently, this combination has become an established method for pre-operative planning and stereotactic neuronavigation in brain tumor patients [7]–[9] because it provides patient-specific information about individual anatomy and displacement effects resulting from tumor growth [7], [10]. Due to brain shift and tumor resection during surgical procedures, shifting of fibers may occur which requires intra-operative tractography to ensure maximum tumor resection and preservation of neurological function [11], [12]. To meet clinical requirements, accurate and fast fiber reconstruction methods that can be categorized into *local* and *global* approaches are required:

Several local approaches based on the principle of fiber tracking such as streamline propagation (SP) [13], tensor deflection (TD) [14] and the FACT algorithm [15] have been devised. They make restricted use of local tensor information by deterministically computing a fiber direction at each considered point and propagate fibers sequentially in independent steps. A general limitation of this concept is that minor inaccuracies in the chain of local decisions can accumulate and significantly affect the final result. Therefore, local approaches have difficulties with crossing or branching pathways or with pathways in regions of low anisotropic diffusion.

In contrast, global approaches consider a larger subspace of the diffusion tensor field at once to infer global characteristics of the diffusion process and to model uncertainty in the diffusion data. For instance, fast marching methods evolve a diffusion front according to the underlying tensor profiles and compute fibers with an assigned level of confidence by backward processing based on gradient descent [16], [17]. Another approach applies Monte-Carlo simulation of fiber tracking to provide a probabilistic interpretation of tensors [18]. Multiple fibers originating from one seed point are created by randomly sampling probability distributions defined on the diffusion tensor. From probability maps computed by counting the number of fibers crossing each voxel, conclusions on the architecture of the neuronal network can be derived. In [19], two connectivity maps are computed for two seed regions of interest (ROIs). Based on the number of fibers that cross each voxel in the same or the opposing direction of the major eigenvector, areas of connecting and merging pathways can be separated. By computing a probability index for connections to both seed regions (PIBS), this approach (denoted in the following as ConProb) enforces the expressiveness of probability maps for structural connectivity as compared to probabilistic tractography using one seed region. Another approach based on particle simulation models the diffusion field by external energies and the location and direction of and connection between short fiber segments by internal energies [20]. Optimized by a Markov chain Monte Carlo method and a tailored local tracking algorithm, this approach (denoted as GibbsT) produces optimally distributed fibers along the white matter structures [21], [22].

Either of these global approaches computes fibers or probability maps of high quality, however, the computational effort is beyond a clinically acceptable time frame. Other global approaches introduce the concept of target-orientation explicitly by reconstructing connections between two predefined ROIs. This reduces the amount of data while the level of quality is maintained. The guided fiber approach [23] extends a standard fiber tracking algorithm by a random sampling strategy that is constrained to a narrow region surrounding the expected location of the pathway. Since multiple alternative directions are considered, complex pathway arrangements in regions of low anisotropic diffusion can be resolved. Even more alternative directions are considered by a search for minimum cost paths that is combined with a Bayesian framework for tensor field regularization [24].

Our previously presented approach [25] applies a global search (GS) to evolve optimal connections between two functional areas according to a diffusion dependent cost function. As this function is sampled for a large number of directions at each point of the search space, complex pathway configurations can be resolved. Moreover, GS is based on the A^*^ shortest path algorithm, whose time complexity is optimal [26], i.e. it is guaranteed that the best possible solution is found with the smallest computational effort. For this reason, GS is particularly suitable for clinical application.

In this work, GS was compared with the aforementioned fiber tracking algorithms, SP and TD, which are commonly used in clinical practice [7], [8]. Two tractography algorithms, ConProb and GibbsT, which employ stronger concepts such as explicit regularization to model noise in diffusion data and global optimization with particle simulation, were additionally included in the evaluation.

To evaluate the reconstruction capabilities of the considered methods, two neuronal pathways, the arcuate fasciculus (AF) and the inferior fronto-occipital fasciculus (IFOF), were selected. These pathways are of particular interest for neurosurgical interventions as they connect the Broca and Wernicke areas by a dual route that is part of the language processing system. The AF, which is a part of the superior longitudinal fasciculus, is generally regarded as the dorsal language pathway responsible for phonological processing [3], [27], [28]. In contrast, the exact anatomical correlate of the ventral semantic pathway is not clearly established. Various fiber tracts have been described, including the inferior longitudinal fascicle (ILF) [3], [29], middle longitudinal fascicle [3] and the IFOF to subserve this connection [29]. The function of the ILF for language processing was recently described as dispensable by a subcortical stimulation study [30], whereas the function of the IFOF was found to be crucial. Moreover, for both the existence of the IFOF in humans and its role in language processing, there is a wealth of anatomical [31], [32], neurosurgical [28], [30], [33], and diffusion tensor imaging studies [34], [35]. The relevance of this tract for semantic processing was additionally confirmed by a study on young adults with dyslexia showing significant correlation with DTI [36]. In a dissection study of brains of rhesus monkey, the existence of this pathway has been challenged [37], but as white matter tracts serving the language processing might be underdeveloped, conclusions following the transition between species might not be strong enough to counter recent research.

Despite of this controversy, the IFOF was selected to challenge the above tractography approaches as its reconstruction in the context of semantic language processing has been shown to be a difficult task. A combined fMRI and DTI study for language relevant cortical sites and white matter pathways of healthy subjects identified problems of fiber tracking to reveal the terminals of the IFOF in the middle temporal gyrus that are relevant for semantic processing [29]. This was explained by the stronger occipital terminals and the tendency of streamline tractography to follow the other major neuronal pathways in this region. Due to this difficulties, assessing the capabilities of GS, SP, TD, GibbsT and ConProb in the context of brain tissue affected by tumors is a challenging research question.

For this purpose, fiber tracts reconstructed by all methods on image data of both patients undergoing tumor surgery and healthy controls were assessed by a neuromedical expert under the qualitative aspect of anatomical feasibility. Then, the agreement of the reconstruction methods on the location of neuronal pathways was evaluated quantitatively by distance- and diffusion-based similarity metrics. In addition, all methods were validated on ground-truth data of simulated DTI software phantoms comprising fiber configurations of varying complexity.

## Materials

### Ethics Statement

This study was approved by the ethics committee of the University of Erlangen-Nuremberg according to the ethics committee proposal number 3578. This included all clinical investigations with pre- and intra-operative MRI and functional neuronavigation, and subsequent data analyses for all patients and controls. Written informed consent was obtained from all patients and controls. All clinical investigations were conducted according to the Declaration of Helsinki.

### Image Data

Image data originated from 25 right-handed patients (11 female, 14 male, with mean age of 41.6
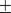
12.6 years) and 6 right-handed controls (1 female, 5 male with mean age of 34.7
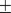
9.9 years, 4 with tumors in right hemisphere, 2 healthy probands), all with left-hemisphere dominance. The two groups did not significantly differ in age (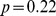 by non-parametric tests of Mann-Whitney [38] with significance level 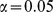). The patient group comprised subjects with primary brain tumors (WHO grade I–IV). To support the grading, the volume of tumors was assessed by manual or semi-automatic intensity-based segmentation. For enhancing tumors, post-contrast T1-MPRAGE scans, acquired as mentioned below, provided images with appropriate contrast. For nonenhancing tumors, images were acquired with T2-weighted scans.

All tumors were located in the white matter of the left dominant hemisphere along the course of the language pathways. Even with larger distances between tumors and neuronal pathways, the influence of tumors can be strong in terms of both fiber displacement and impairment of associated functions. Since functional deficits were observed also in these cases, patients were included with distances from 1 up to 30 mm. The controls were considered to assess the similarity of fiber tracts reconstructed with different methods in unaffected brain tissue.

For all subjects, the image data was acquired between 2005 and 2009 with a Magnetom Sonata Maestro Class 1.5 Tesla scanner (Siemens Medical Solutions, Erlangen, Germany) equipped with a gradient system with a field strength of up to 40 mT/m (effective 69 mT/m) and a slew rate of up to 200 T/m/s (effective 346 T/m/s).

#### Data Acquisition – fMRI

During the acquisition of functional data using fMRI, visual stimulation was performed because of the reduced signal-to-noise ratio as compared to auditory stimulation. For each subject, 30 rest and activation measurements were obtained according to a block paradigm (“boxcar”) with a total duration of 15 minutes. Activities included reading, naming of objects, answering questions and inferring verbs and sentences from given nouns [9].

The functional data was acquired using an echo-planar imaging (EPI) sequence (parameters: TR = 2470 ms, TE = 60 ms, image matrix 64
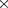
64, slice thickness 3 mm, resolution 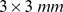, 16–25 slices). These measurements were interpolated to 75 slices with 1 mm in plane resolution. An image based prospective acquisition correction for head motion was used [39]. Hereby, interpolation was done in the k-space.

Statistical functional mapping on a voxel-by-voxel basis of activated functional areas was performed using BrainVoyager [2]. Activation maps were individually determined by analyzing the correlation for each voxel between signal intensity and a square wave reference function according the paradigm. Voxels exceeding a threshold (typically 0.40) were displayed if at least six contiguous voxels of the measured slices built a cluster to eliminate isolated voxels. The functional slices were aligned to the MPRAGE images with 160 slices of 1 mm slice thickness and resolution obtained in the same patient position.

According to this mapping, areas associated with language functions were typically located in the frontal and temporo-parietal lobes of the left hemisphere (right-handed population) that correspond to the Broca and Wernicke areas. These areas could be determined for all subjects successfully and were selected as ROIs for all tractography approaches.

#### Data Acquisition – DTI

For the DTI measurements, a single-shot spin-echo diffusion weighted EPI sequence was used (parameters: TR = 9200 ms, TE = 86 ms, b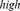 = 1000 s/mm^2^, b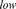 = 0 s/mm^2^, field of view 240 mm, 1500 Hz/Px bandwidth, measurement time: 5 min and 31 sec). For each slice, five measurements were acquired and averaged by the MRI scanner. The image resolution was 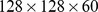 voxels with a respective voxel size of 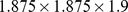 mm^3^. One reference image without diffusion sensitization as well as six images with diffusion sensitization in non-collinear directions were obtained according to the oblique double gradient encoding scheme ((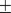
1,1,0), (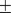
1,0,1), and (0,1,
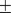
1)) [40]. Compared to numerically optimized gradient directions, this scheme provides relatively low error rates for the computation of tensors and FA values, but a slightly higher sensitivity to measurement noise [41]. Moreover, compared to acquisitions using 30 diffusion gradient directions, the influences on diffusion values and tractography results are within an acceptable range [42].

#### Data Acquisition - Anatomical MRI

Anatomical data was acquired for pre-operative planning, intra-operative neuronavigation and anatomical validation by a 3D gradient echo sequence MPRAGE (Magnetization Prepared Rapid Acquisition Gradient Echo) (parameters: TR = 2020 ms, TE = 4.38 ms, field of view 250 mm, 130 Hz/Px bandwidth, measurement time: 8 min 39 sec) with a resolution of 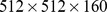 voxels and a respective voxel size of 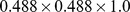 mm^3^.

Additionally, if the tumor exhibited contrast enhancement on the preoperative images, the T1-weighted axial spin echo sequence was repeated after intravenous application of contrast media (0.2 ml/kg gadolinium diethylenetriamine penta-acetic acid). In nonenhancing tumors, an additional T2-weighted turbo spin echo sequence (parameters: slice thickness 4 mm, field of view 230 mm, TR = 6490 ms, TE = 98 ms, scan time: 5 min 59 sec at three acquisitions) was acquired.

### Simulated Data

To validate the fiber reconstruction methods on ground-truth data, three DTI software phantoms were selected from a database with fiber configurations of different complexity including branching and kissing pathways as well as a spiral with more than three turns [43]. While the character of branching and kissing pathways is similar to major pathways such as the pyramidal and optical tracts, strong curvatures are typical for pathways with cortical terminations such as the language pathways along the AF and IFOF.

According to a study on the influence of signal-to-noise ratio (SNR) on the pathway reconstruction [44] that used a similar scanner type and DTI protocol, SNR varies between 15 and 25. Therefore, phantom images were selected with a simulated SNR of 15 and 30. To allow for a direct comparison of similarities between reconstructed tracts, the voxel size was adjusted to comply with the image data.

## Methods

### Global search (GS)

The approach of GS to anatomical connectivity [25], that is compared in this study with SP, TD, GibbsT and ConProb, searches for minimum cost paths by evolving a search tree on a structured grid between two functional areas identified as ROIs by fMRI. The cost function originally minimized in [25] is based on the propagation probability function 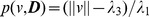 with 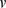 denoting the current propagation direction and 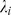 being the eigenvalues of 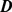 in descending order. To prevent a bias towards isotropic tensors, the isotropic fraction 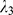 is subtracted, while the shape of the probability profile is still maintained. The cost for each path considered by the A^*^ shortest path algorithm is then defined by accumulating the negative proportional function 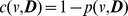 over all path segments.

In this work, this cost function is extended in two ways: At first, the divergence 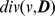 from the principal diffusion direction (Eq. 1) is incorporated to improve guidance through regions of low anisotropic diffusion close to functional areas and tumor infiltrated tissue. Since the principal diffusion direction is not well-defined in isotropic tissue, the geometric shape of tensor ellipsoids [45] has to be considered. For linear ellipsoids, divergence is defined as the cosine of the angle between 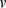 and the first eigenvector 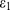, whereas for planar and spherical ellipsoids the cosine of the angle between 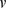 and the plane spanned by the first two eigenvectors is considered. The second extension includes fractional anisotropy 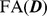 (Eq. 2) [6] to support guidance in anisotropic regions. All metrics are then combined into a new cost function 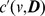 (Eq. 3).
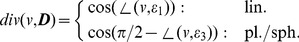
(1)

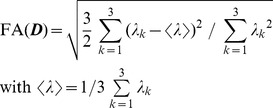
(2)

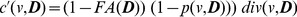
(3)


The cost function is additionally supported by thresholds for the maximum bending angle between adjacent fiber segments and the minimum FA value at sample points. Thereby, the reconstruction of anatomically unlikely fibers of strong curvature or of fibers intersecting isotropic regions can be avoided. Moreover, anatomical knowledge can be incorporated to reconstruct specific pathways by restricting the search space to an adjustable bounding box. This is especially useful for the dual route of the language system, since anisotropic diffusion is different along the AF and IFOF [46] and may also depend on the location of the tumor [10].

### Deterministic fiber tracking

The algorithms SP and TD evaluated in this study are clinically approved and implemented in various DTI applications [7], [8]. Fiber tracking is initialized at seed points with sufficiently high FA value selected from the whole volume. From the resulting set of fibers, only those that intersect both ROIs are kept. In this way, propagation becomes more robust, as it is not initialized in functional areas with unclear diffusion direction, but in regions of anisotropic diffusion referring to white matter.

Fibers are propagated in both ortho- and retrograde directions and are terminated as soon as the FA value drops below a predefined threshold. To propagate fibers from the current location, different schemes are applied by SP and TD: In case of TD, the diffusion tensor is multiplied with the fiber direction of the previous fiber segment to obtain the next fiber point. In regions of high anisotropy, fibers are deflected towards the major eigenvector, whereas in isotropic regions, the incoming direction is maintained or only slightly affected by the tensor [14]. In contrast, fiber tracking with SP applies the 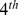-order Runge-Kutta streamline integration scheme that linearly combines the major eigenvectors of the interpolated diffusion tensors at four sample points to determine the next fiber point [13].

### Similarity metrics

Several similarity metrics for reconstructed fiber tracts were proposed in the literature, which are either applied to cluster fibers or to evaluate approaches for pathway reconstruction by comparing the divergence of fibers from ground-truth data or from the results of other methods. In general, the similarity of two fibers can be quantified by their spatial displacement [21], [47]–[50], by the angular deviation of a direction [21], or by geometric shape properties such as curvature [21], [50]. Since SP, TD and GibbsT inherently generate smooth fibers, whereas fibers of GS are grid-bound, assessing the curvature or angular deviation would always provide favorable results for the former methods. Therefore, the similarity metric 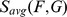 presented in this section is based on the distance between fibers.

#### Geometric similarity

The spatial similarity of the resulting fiber tracts 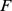 and 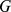 of two methods, defined as 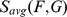 (Eq. 4), measures their deviation by averaging distances among a set of closest fiber pairs defined by relation 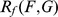 (Eq. 6). The set contains all fibers in 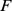, each one paired with the closest fiber in 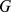, and vice versa, whereas closeness is measured in terms of the similarity metric for fiber pairs 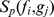 and the trimming method 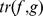 presented later on. Since duplicate fiber pairs are avoided by 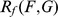, the effect of outliers is emphasized as compared to using all fiber pairs. In addition to the averaging metric 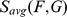, 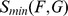 (Eq. 5) determines the closest fiber pair.
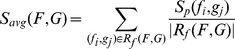
(4)

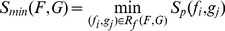
(5)

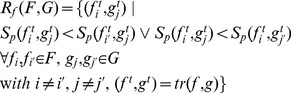
(6)


To measure the distance between two fibers 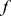 and 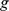, the relation of closest points 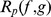 (Eq. 7) assigns to each point 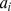 on fiber 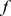 the closest point 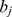 on fiber 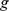 and vice versa. The mean of closest point distances 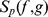 (Eq. 8) is then defined as the average distance between these closest points with 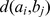 denoting the Euclidean distance 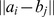. In contrast to the mean of closest point distances defined in [50] that creates symmetry by averaging two similarity values of a bi-directionally applied non-symmetric metric, 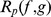 excludes duplicates from the resulting set of closest points to emphasize outliers.
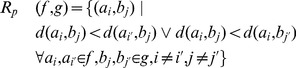
(7)

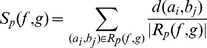
(8)


#### Normalization of fiber pairs

Prior to applying the similarity metric 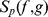 to every fiber pair considered by 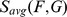, two normalization steps are carried out to account for two geometric differences in the results obtained by GS, SP, TD and GibbsT. Thereby, the trimming method 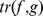 reduces each fiber pair 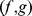 to 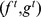.

While both deterministic fiber tracking approaches propagate in arbitrary directions with constant step length, fibers of GS are grid-bound with varying step length and fibers of GibbsT are constructed from points with arbitrary distances. Therefore, fibers of GS and GibbsT are resampled first to reduce the deviation of point sets introduced by the different propagation methods. This is achieved by replacing the original points of the fibers of GS by points of equal distance corresponding to the constant step length of SP and TD. In contrast to the smoothing of fibers with B-splines in [21], this normalization approach does not spatially modify the results.

**Figure 1:**
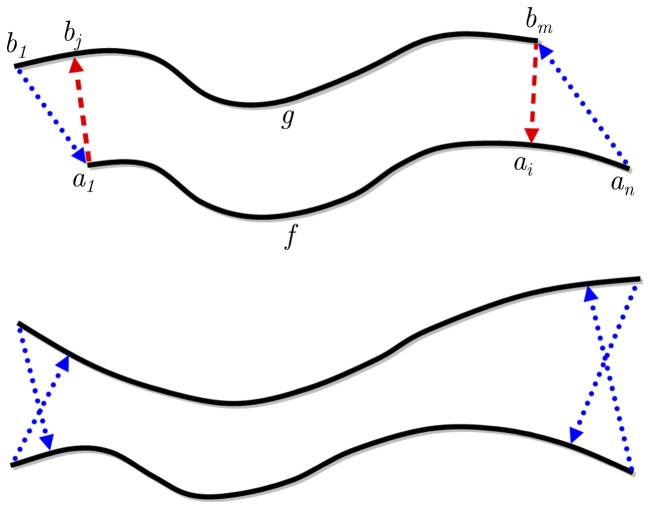
Trimming of fibers with closest point pairs at fiber endings. *Top*: Trimming of overlapping endings according to [47]. Segments between end points and intermediate points are removed (end points to dashed lines). *Bottom*: Trimming of diverging fibers is avoided with the new approach (end points to dotted lines).

Secondly, overlapping fiber segments have to be identified to avoid biasing of the similarity metric, as fibers of GS terminate in ROIs, whereas fibers of SP, TD and GibbsT are not restricted by them. For two fibers 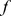 and 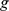 selected from two fiber tracts 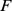 and *G*, the overlapping segments 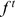 and 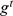 are determined similarly to the trimming approach in [47]. At each end of the fibers, the closest point pairs are considered as illustrated in Figure 1 *(top)*. If the first point of these closest point pairs is denoted as 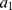, the second point is either the end point 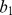 or an intermediate point 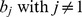 depending on which fiber overlaps the other. If the second point is an intermediate point, the protruding segment between 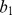 and 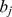 gets removed. The same is repeated for all closest point pairs on either side of the two fibers.

This approach is suited to cut protruding endings of otherwise coherent fibers, as the overall structure of the fibers is not altered. However, in case of diverging fibers as illustrated in Figure 1 *(bottom)*, more fiber segments than necessary would be excluded. Therefore, the trimming approach was extended as follows: Again, the two closest point pairs at each end are considered, but trimming is applied to at most one of the fibers at each end under the condition that only one of the closest point pairs refers to an intermediate point, while the other refers to an end point.

#### Diffusion similarity

In addition to geometric properties, similarity in terms of diffusion is evaluated for each pair of fiber tracts as 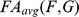 (Eq. 9) to reveal differences between groups, methods and pathways. For both fiber tracts 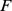 and 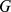, the average FA values are computed for their trimmed and correlated fibers according to
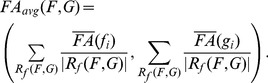
(9)


## Experimental Design

For detailed assessment of the capabilities to reconstruct anatomically feasible fiber tracts in the presence of nearby tumors, the clinically approved methods SP and TD, and the global approaches GibbsT and GS were compared with each other using the similarity metrics defined above. These methods were additionally compared with ConProb by visually assessing the overlap of the fibers with the probability maps.

All methods were applied in native space for direct comparison. Fiber tracking in standard space, provided by a registration method capable to deal with tumor data, was not applied because this study is intended to provide results of direct relevance for state-of-the-art neurosurgery.

After an evaluation of SP, TD, GibbsT and GS using DTI software phantoms, all methods were applied on two pathways of the language system, the AF and the IFOF. Results for the patient and control group were considered separately to investigate the influence of nearby tumors. Each pair of methods was evaluated in terms of the presented similarity metric 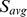 and the average FA value 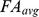. To highlight the challenges that each of these reconstruction problems pose, both, the two pathways and the two groups, were considered separately. By applying non-parametric tests of Mann-Whitney [38] with a significance level of 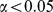, statistical significance was tested for the defined groups.

Details about the parameters and adjustments to the connectivity problems for either method are presented in the following: Generally, for both the simulated and the image data, the step length of SP and TD was set to 0.5 mm. The search space of GS was defined by a cubic grid with 74 neighboring points and an average step length of GS to 2 mm, resulting in a smallest and largest step length of 1.3 and 2.3 mm, respectively. The threshold on the bending angle of GS was set to 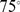. All methods sampled the diffusion tensors at arbitrary locations by trilinear interpolation.

For the software phantoms, ROIs covering the full diameter of the fiber tracts were manually positioned at both ends of the simulated pathways. As the diffusion characteristics were different for each DTI phantom, FA thresholds were defined individually and set to 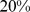 below the average FA value in the main part of the pathways for all methods. Restriction of the search space using a bounding box was not applied for the simulated data.

For the experiments based on image data of patients and healthy controls, ROIs corresponding to the speech areas obtained by fMRI were defined. The fibers created by SP and TD were separated into the dorsal and the ventral pathway by two manually defined ROIs placed at the middle of each tract.

Due to the aforementioned drawbacks of deterministic fiber tracking methods in regions of low FA, they may not succeed in locating a connection between these ROIs. Therefore, two modifications to the parameters and ROIs were applied: At first, tracking was performed with FA thresholds of 0.25 and on failure with 0.2. Secondly, ROIs were incrementally expanded by at most 4 voxels or 7.6 mm in each dimension until a connection was found with either FA threshold. In the following, this process is denoted as ROI padding. The decision rule about which fiber tract to select for the similarity metric was defined as follows: If fiber tracking with an FA threshold of 0.25 required no more than two additional ROI paddings as compared to fiber tracking with an FA threshold of 0.2, the fiber tracts with FA threshold of 0.25 were used. Otherwise, the fiber tracts with FA threshold of 0.2 contributed to the similarity value.

GibbsT was applied using the MITK implementation as presented in [51]. Given only six gradient diffusion data sets, the approximation of the orientation distribution function (ODF) with 252 directions was accomplished using spherical harmonics of order 4 according to [52]. As the ODFs are based on far less diffusion directions as usually required for high angular resolution diffusion imaging (at least 30), resolving complex fiber arrangements as in [20] might not be possible. Nevertheless, GibbsT based on the major diffusion direction is expected to produce fibers of at least the same quality as deterministic fiber tracking.

The fiber reconstruction itself was applied using the following parameters: 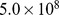 iterations, start and end temperatures of 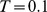 and 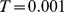, 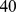 mm minimum fiber length, equally weighted internal and external energies, a curvature threshold of 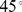 and subtraction of the ODF mean. The particle parameters were defined as length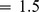 mm, width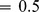 mm and weight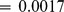. Fibers of GibbsT were imported into MedAlyVis (Medical Analysis and Visualization, Computer Graphics, University of Erlangen, Germany), the platform for generation of fiber tracts of SP, TD, and GS, and their visualization and comparison. Similarly to SP and TD, GibbsT applies fiber tracking to the whole brain and tends to generate fibers not reaching both ROIs. Therefore, ROI padding was applied as well.

Regarding ConProb, connection probability maps for both ROIs (without padding) were generated in the Matlab-based DTI & FiberTools [53] using 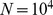 randomly computed fibers with a maximum length of 150 voxels and an exponent of 4 on eigenvalues to sharpen diffusion tensors. The resulting two probability maps were combined to the “probability index of forming part of the bundle of interest” (PIBI) as described in [19], and imported into MedAlyVis for visual comparison.

In order to search for anatomical connections with GS, the bounding box was manually placed for both the AF and IFOF by considering anatomical features and activation centers. In case of the AF, the bounding box was placed to include the white matter of the left hemisphere without the rostral part of the temporal lobe. Regarding the IFOF, the upper boundary of the bounding box was placed above the frontal horn of the lateral ventricle, and the inner boundary directly medial to the external capsule. In all cases, the FA threshold was initially set to 0.3 and reduced to 0.15 in case of failure. In contrast to SP, TD and GibbsT, ROI padding was not applied for GS.

**Figure 2:**
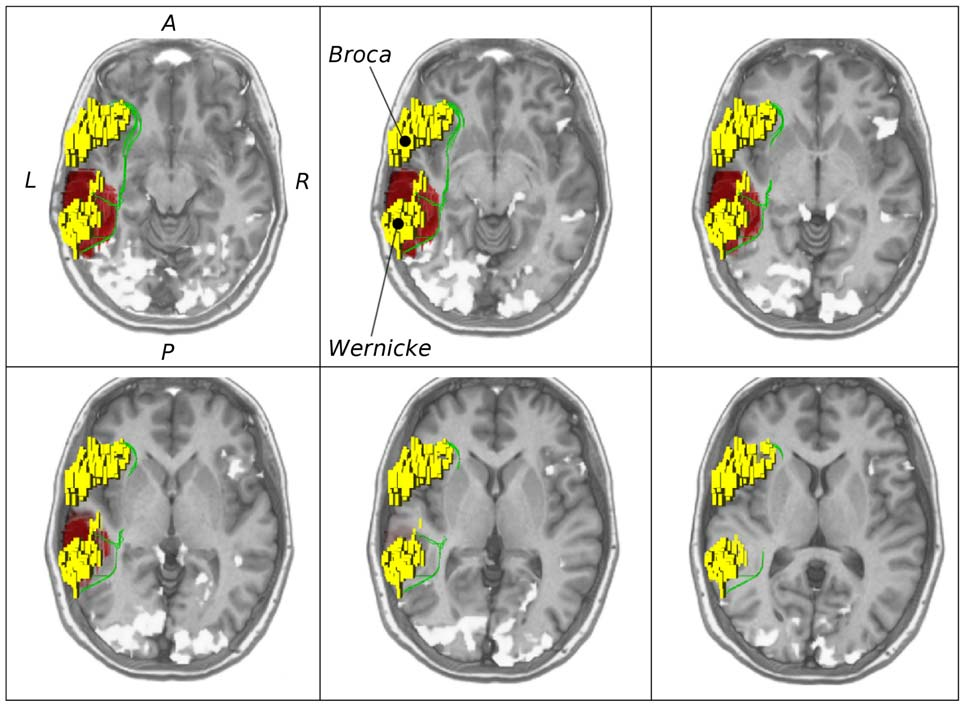
Validation on medical data. Axial slices of MRI data in upward order with the Broca and Wernicke speech areas (yellow), fMRI activations (white and yellow) and ventral pathways along the inferior fronto-occipital fasciculus (IFOF) reconstructed by global search (GS) (green). Images show patient (female, 36 years old) with language lateralized to the left hemisphere and with a left temporal astrocytoma (WHO grade II, shown in red).

Prior to statistical evaluation, each pair of reconstructed fiber tracts was inspected visually to ensure that the expected anatomical space of the language site is covered. For this purpose, the neuromedical expert (coauthor A. Z.) investigated slices of the T1 MR images with an overlaid display of fiber tracts, fMRI activation clusters, and tumor segmentation (if applicable) as illustrated in Figure 2. Pairs of fiber tracts were excluded from the similarity evaluation if at least one of the fiber tracts did not cover main parts of the AF or the IFOF.

## Results

The evaluation of the methods on ground truth data using DTI phantoms is presented in the next section. The results of the evaluations carried out on the controls with unaffected brain anatomy are explicated in the second section, on the brain tumor patients in the third section.

### Evaluation on simulated data

**Figure 3:**
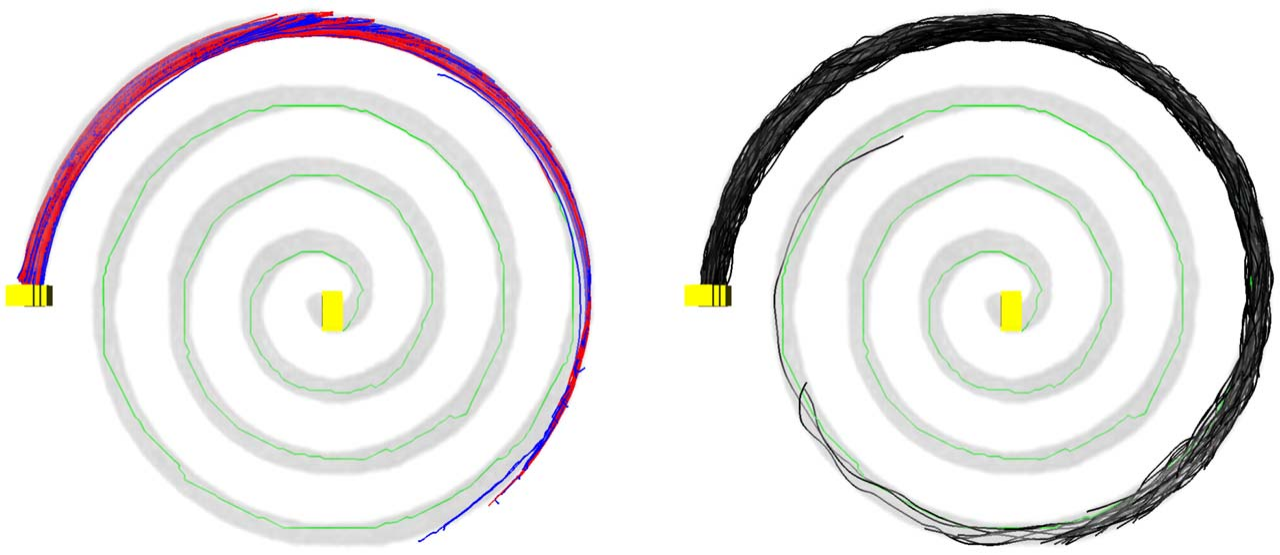
Fibers on spiral DTI phantom with signal-to-noise ratio (SNR) of 30. Left: GS (green), SP (blue), and TD (red). Right: GS (green), GibbsT (black). Simulated pathway highlighted by the first gradient image (gray).

For a qualitative evaluation on simulated data, success and failure to connect the ROIs was assessed in a first step. All methods successfully reconstructed branching and kissing pathways for images with both SNR 15 and 30, leading to similarity results. In case of the simulated spiral, only about two thirds of the first cycle was covered by SP and TD before the fibers terminated at the border of the pathway (Figure 3). Fiber segments generated by GibbsT covered the whole spiral, however connected fibers achieved not much more than one cycle. In contrast, GS successfully reconstructed a trajectory along the full extent of the spiral.

Based on the previously defined similarity metric 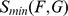 and images with SNR 30, the deviation between fiber tracts of SP and TD amounted to 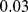 mm in the branching case and 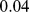 mm in the kissing case. The deviations between SP and GS on the same phantoms were 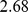 mm and 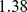 mm, respectively. The alignment between SP and GS could be improved by increasing the angular resolution of the grid. Using a cubic grid with 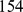 neighboring points and an average step length of 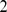 mm, these similarity values amounted to 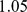 mm and 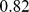 mm. As a result, the course of the closest fibers was almost identical for both methods. Regarding GibbsT compared with GS, deviations of 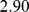 mm and 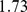 mm were observed in the branching and the kissing case, respectively.

### Evaluation of control subjects

#### Reconstruction of pathways

On image data of control subjects, both SP and TD were able to reconstruct neuronal pathways, although relaxation of the FA threshold and incremental ROI padding had to be applied in some cases to obtain fibers. In one case, the FA threshold had to be reduced to 0.2 for the ventral pathway. For the remaining fibers along the ventral and all fibers along the dorsal pathway, an FA threshold of 0.25 was sufficient. The ROIs were padded on average by 0.17 and 0.67 voxels for the dorsal and ventral pathways, respectively. GibbsT established dorsal and ventral connections in all cases with an average ROI padding of 0.17 and 3.33 voxels respectively. Similarly, GS could establish dorsal and ventral connections in all control subjects with an FA threshold of 0.3.

#### Visual inspection

**Figure 4:**
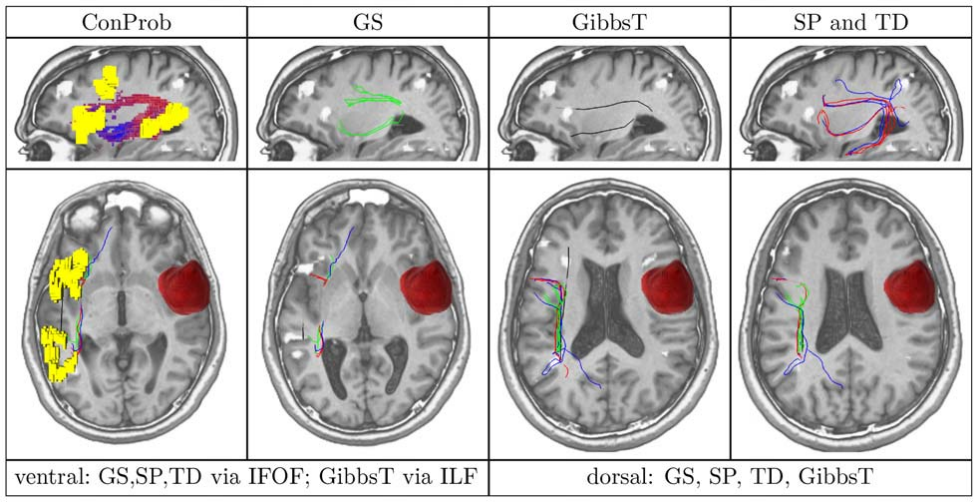
Overview of ConProb, fibers of GS, GibbsT, SP and TD for control patient (male, 36 years) with fronto-lateral anaplastic astrocytoma (WHO grade III) in the right hemisphere. *Upper row* (sagittal views): Image 1: Connection probability map (low to high probabilities from blue to red), Images 2–4: dorsal and ventral fibers of GS (green), GibbsT (black), and of SP (blue) and TD (red). *Lower row:* Axial consecutive images (view from top), two for ventral fibers, and two for dorsal fibers. Tumor segmentations rendered in red. fMRI activations in Broca and Wernicke speech areas (yellow). The following Figures 7 to 10 are arranged in the same way.

After visual inspection, the fiber tracts generated by all methods along the dorsal pathway were confirmed to follow the AF anatomically in all cases of the control group. Regarding the match between the ventral fibers and the anatomy of the IFOF, GS was successful in all cases, while SP and TD established connections along the correct pathway in five and three cases, respectively. In the remaining cases, at least one of the methods reconstructed fibers corresponding to the ILF. Similar results were created by GibbsT for the ventral fibers. Here, fibers did not match the IFOF, but the ILF for all cases which is exemplified for the control patient in Figure 4. From visual comparison of the PIBI maps by ConProb, a strong agreement between ConProb and GS on the anatomy of the dorsal and the ventral pathway was found which is highlighted in Figure 4. The AF and the IFOF were found in all cases, whereas pathways corresponding to the ILF could be established in two cases only.

#### Similarity metrics

**Figure 5:**
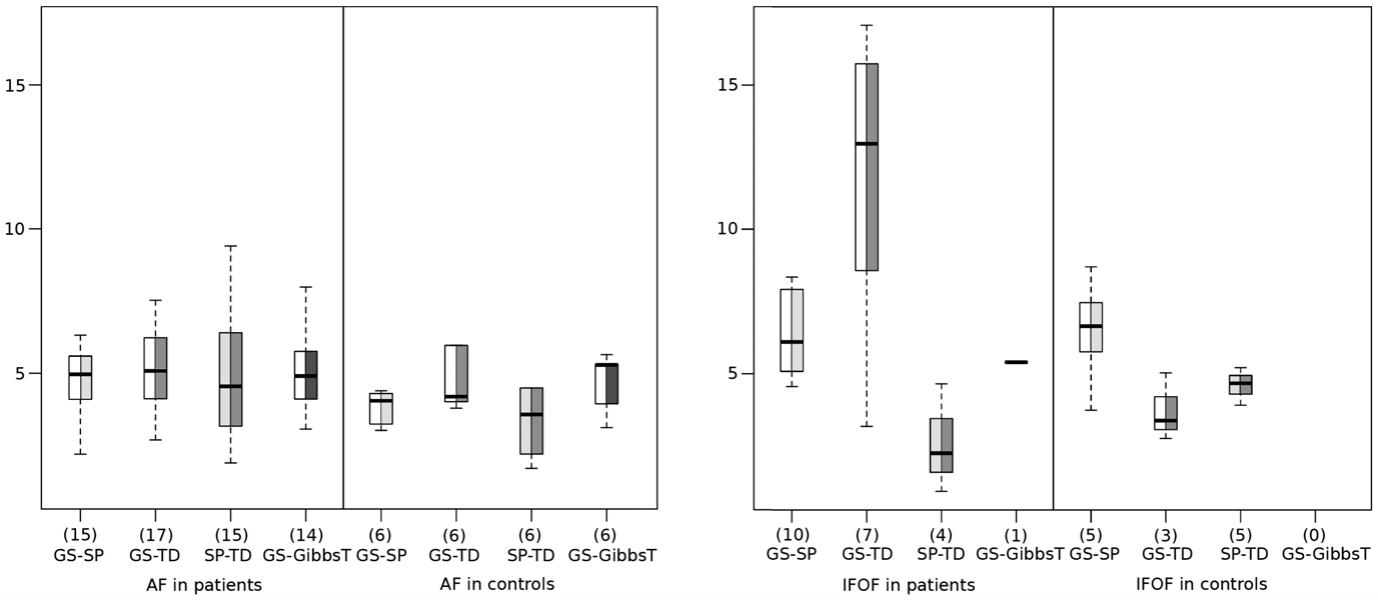
Boxplots showing the average of similarity measure 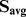 in mm for the arcuate fasciculus (AF) (left) and the inferior fronto-occipital fasciculus (IFOF) (right) in the patient and control group for each of the compared pairs of methods. The numbers N of matching fiber tract pairs are provided in parenthesis. These are reduced compared to the numbers of patients (25) and controls (6) as not all of the reconstructed fibers corresponded to anatomical feasible structures.

Results of pairwise comparison between all the methods excluding ConProb are summarized in the boxplots in Figure 5 with similarity metrics 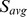 averaged for corresponding fiber tracts. 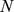 represents the number of pairs of fiber tracts that cover the same anatomical space visually identified as either AF or IFOF.

For the control group, complete agreement of all methods was found for the AF, whereas less matching pairs between GS and SP and between GS and TD were found for the IFOF, and none for GS and GibbsT. Regarding the geometric similarities along the AF, fiber tracts of GS and SP were on average closer than of GS and TD and of GS and GibbsT. Along the IFOF, fiber tracts of GS and TD were closer than those of GS and SP. For GibbsT, no fiber was found for the IFOF.

**Figure 6:**
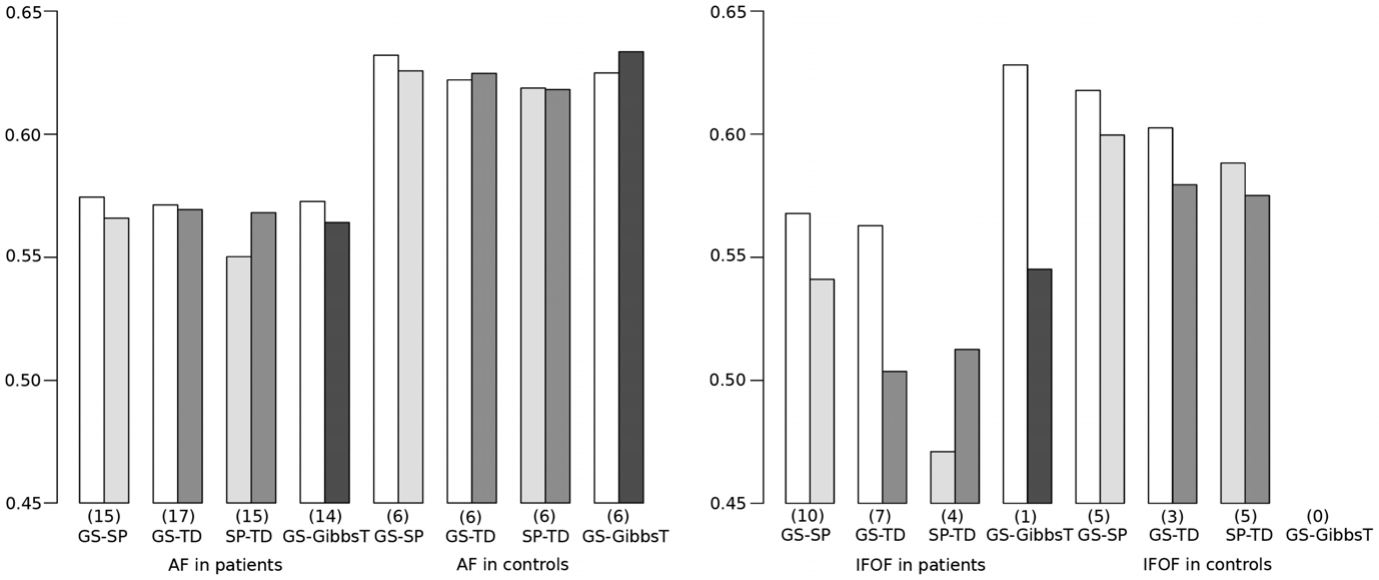
Barplots showing the average fractional anisotropy (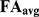) of the resulting fiber tracts of each compared pair of methods for the AF (left) and IFOF (right) in the patient and control group. The numbers N of matching fiber tract pairs are provided in parenthesis. For an explanation of the reduced numbers see Figure 5.

To elucidate the effect of methods on FA, values of 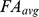 averaged across subjects are summarized in the barplots in Figure 6 for the AF and the IFOF reconstructed with different methods for both groups. Regarding the different pathways in the control group, larger average FA values were observed for the AF as compared to the IFOF (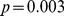). No clear differences could be observed for the methods.

### Evaluation of patient data

#### Reconstruction of pathways

In the patient group, SP and TD depended more on the relaxation of the FA threshold and incremental ROI padding. In all but four cases, an FA threshold of 0.25 enabled reconstruction along dorsal and ventral pathways, in the remaining cases a relaxation to 0.2 was required for both pathways. The average paddings by SP and TD were 1.0 and 0.81 (at most 3) voxels for the dorsal, and 1.63 and 1.33 (at most 4) voxels for the ventral reconstruction. GibbsT established dorsal and ventral connections in 14 and 11 cases with an average ROI padding of 2.07 and 3.09, respectively.

GS reconstructed the dorsal phonological pathway in all but four patients with an FA threshold of 0.3. The remaining cases had either tumors of WHO grade III or IV, or the distance between pathways and the tumor was smaller than 5 mm. By reducing the FA threshold to 0.15, connections could be found in these cases as well. For the ventral semantic pathway, fiber tracts were found by GS in all but two patients with an FA threshold of 0.3. In the remaining cases, patients with a WHO grade IV tumor in the left temporal lobe and a WHO grade II tumor in the temporal stem, GS applied with an FA threshold of 0.15 found no fiber tracts.

#### Visual inspection

After visual inspection, the fiber tracts along the dorsal pathway were confirmed as anatomically feasible in the majority of all patients for SP, TD and GibbsT: 15, 17 and 14 out of 25 cases with ROI padding averages of 1.0, 0.81, and 1.64 respectively. The results of GS were confirmed to follow the AF for all subjects in the patient group.

**Figure 7:**
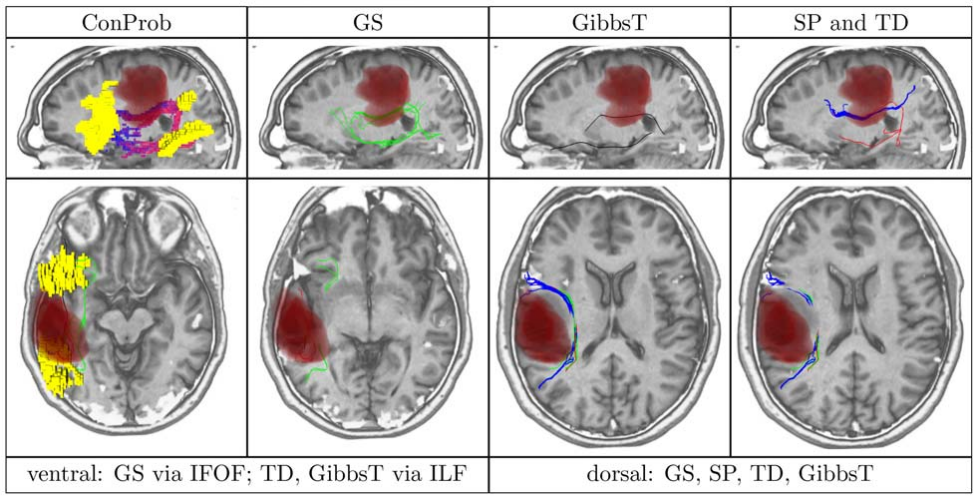
Patient (male, 38 years) with left fronto-parietal astrocytoma (WHO grade II).

**Figure 8:**
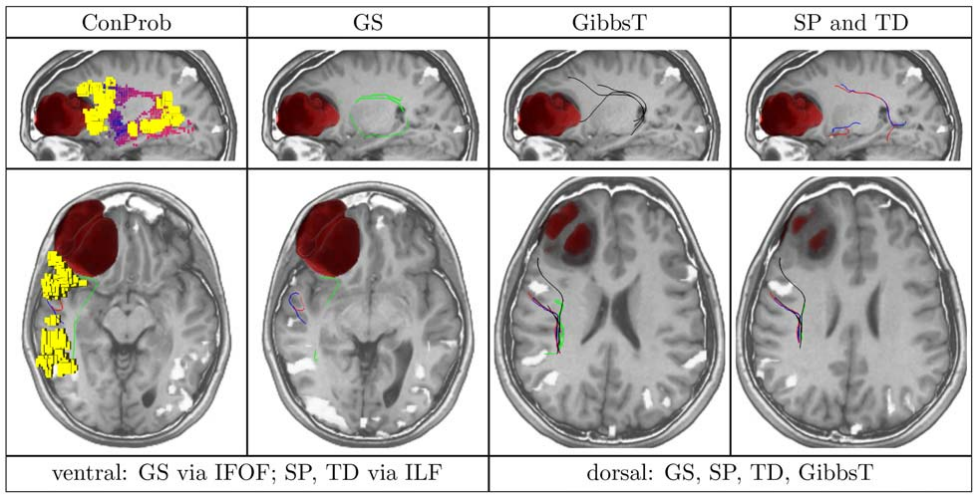
Patient (female, 48 years) with left frontal anaplastic astrocytoma (WHO grade III).

**Figure 9:**
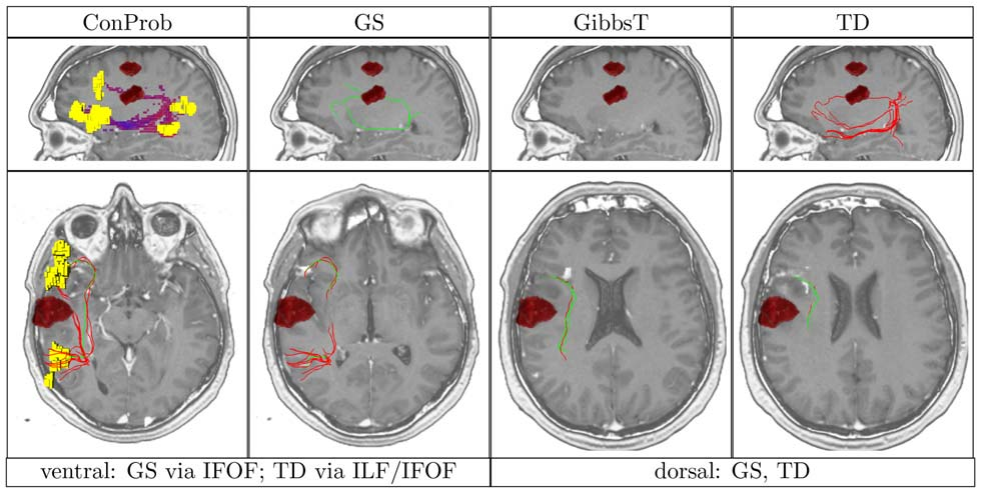
Patient (male, 55 years) with left frontal anaplastic oligodendroglioma (WHO grade III).

**Figure 10:**
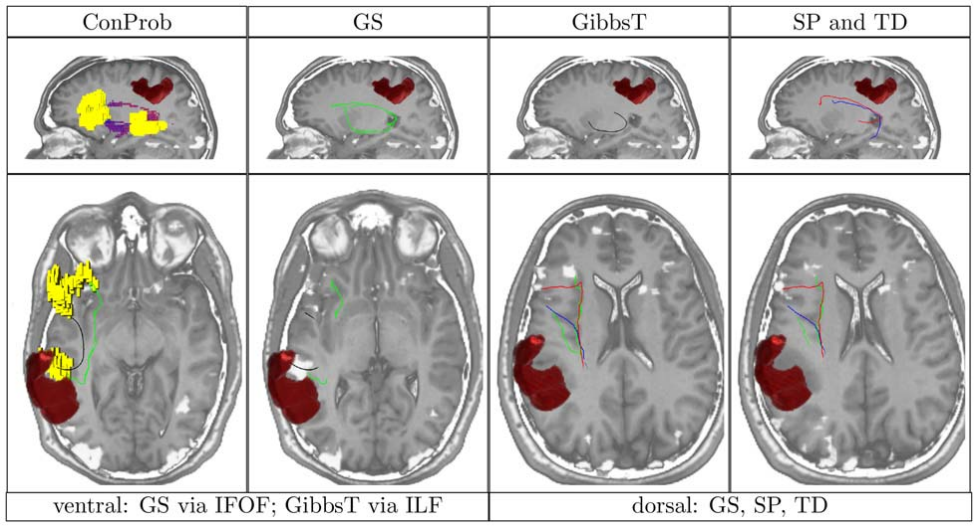
Patient (male, 29 years) with diffuse left post-central astrocytoma (WHO grade II).

**Figure 11:**
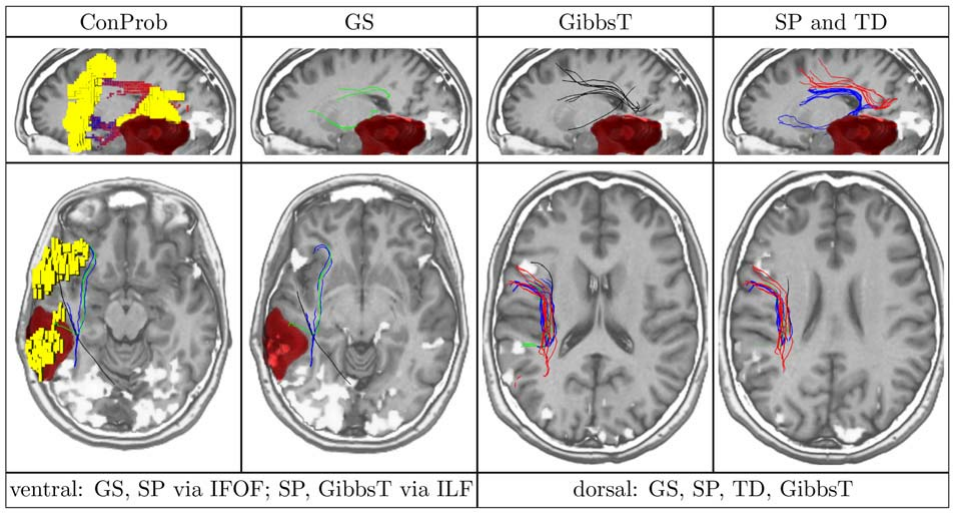
Patient (female, 36 years) with left temporal astrocytoma (WHO grade II).

**Figure 12:**
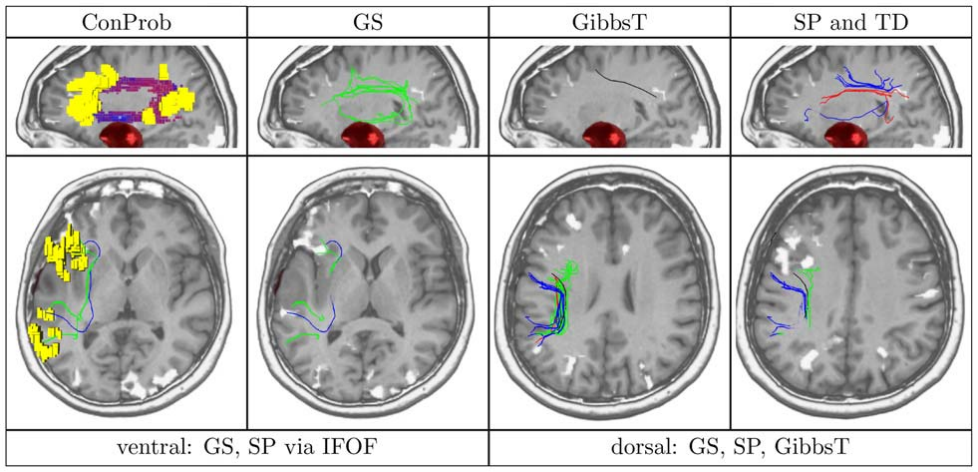
Patient (female, 59 years) with left temporal anaplastic astrocytoma (WHO grade III).

In contrast to the positive results obtained for all methods dorsally, fiber tracts reconstructed by SP and TD along the ventral pathway correlated in some cases with the ILF rather than the IFOF. This problem becomes apparent in Figures 7, 8, 9 and 10 where either SP, TD or both fail to reconstruct the IFOF. In Figures 11 and 12, SP agreed with GS on the IFOF.

By replacing the frontal ROI with the rostral part of the external capsule, the focus of SP and TD could be changed to the IFOF. However, even after this modification, only some fiber tracts with anatomical correspondence to the IFOF could be reconstructed. Regarding the anatomic feasibility of the results of GS, all successfully reconstructed fiber tracts followed the trajectories of the IFOF.

For only one case, ventral fibers of GibbsT followed the IFOF (ROI padding of 3), and the ILF in eight cases (average ROI padding of 2.7) which is shown for the patients in Figures 4, 7, 10, and 11. Regarding the PIBI maps shown in Figures 4, 5, 6, 7, 8, 9, 10, 11 and 12, the dorsal pathway was assigned to the AF in all cases except for two subjects both with a left glioblastoma multiforme (WHO grade IV) located between Broca and Wernicke areas. The ventral pathway corresponded to the IFOF in all but two cases, whereas the ILF was marked in all but nine cases.

#### Similarity metrics

Apparently, when observing the results in Figure 5, there is a higher correspondence and increased geometric similarity for fiber tracts along the AF than along the IFOF when GS is compared with either deterministic fiber tracking method. Across all fiber reconstruction methods, significantly lower values of 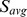 were observed for AF than for IFOF (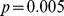).

Regarding the comparison of diffusion values, higher average FA values were observed along the AF than along the IFOF in all compared fiber tract pairs except for the pair GS and GibbsT (compare barplots in Figure 6. Across all methods, a p-value of 0.005 was observed for the patient group, whereas significant differences belonged especially to the pairs GS-TD (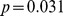) and SP-TD (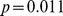).

In case of the AF, visual assessment revealed that the increased diameter of this pathway compared to the IFOF causes a stronger deviation between the methods despite their agreement on the anatomical space of the AF.

### Group differences

Regarding differences of 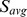 between groups, no statistically significant effects could be established. In the controls, slightly lower values were observed in case of the AF for all compared methods, and in case of the IFOF for GS compared with TD. However, this trend is too weak to show statistical significance. In terms of group effects for diffusion values, significantly reduced average FA values were detected in the patient group in comparison to the controls (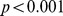). In addition, the average FA values were higher for GS than for SP and TD (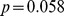), whereas no preference between SP and TD (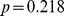) could be observed.

### Computational aspects

All methods were executed on a Intel Pentium Core i7 (3.0 GHz, 12 GByte RAM) and timings were recorded for each subject including loading of data and ROIs, preprocessing such as computation of the diffusion tensors, execution of the methods and automatic filtering with ROIs. SP and TD required between 5 and 10 minutes, and GS took 20 minutes on average. The computation of the connection probability maps amounted to between 15 and 45 minutes. In contrast, the optimization of the Gibbs tracking approach took about two hours.

## Discussion

In this study, five different methods for reconstructing neuronal pathways of the language system were compared: GS and GibbsT as representatives for global approaches, two clinically applied fiber tracking techniques, namely SP and TD, and ConProb, a connection-oriented probabilistic tractography approach.

For the simulated DTI data, GS was able to obtain valid fiber tracts for all configurations, whereas SP, TD, and GibbsT failed to determine connecting fiber tracts in case of the spiral. This clearly indicates the methodological advantages of GS for connectivity problems with known start and end regions. The relatively large spatial deviation between GS and SP or TD can be explained by the grid dependency of GS that becomes obvious as the alignment is improved with grids of higher angular resolution.

Regarding the pathways in the image data, smaller numbers of corresponding pairs of fiber tracts and generally higher distance values were observed for the IFOF as compared to the AF. In addition, average FA values along the IFOF were significantly lower in comparison to the AF. This explains the weaker agreement between the considered methods on the location of the IFOF, and implies that this pathway is more difficult to reconstruct. An additional explanation for these difficulties might be a lower diameter of this pathway and the associated risk to lose track more easily.

In general, a stronger agreement between methods was observed in controls compared to the brain tumor patients as shown by fewer excluded fiber tract pairs. These results clearly indicate the influence of tumors on white matter tracts, which is also reflected by smaller FA values in the presence of nearby tumors. This is a possible reason for the more complicated reconstruction of feasible fibers in brain tumor patients, which is also in line with the detailed analysis of the influence of tumors on FA values in [10]: Accordingly, in white matter infiltrated by tumors or affected by the peritumoral edema, decreased FA values can be observed, whereas displacement and compression of pathways may lead to increasing FA values along pathways. In general, neurological diseases are accompanied by a reduction of FA values. Additionally, the slightly higher mean age of the patient group compared to the control group may lead to decreased FA values [46].

With respect to methods, the application of probabilistic fiber tracking [19] to both groups provided additional information about the structural connectivity problem of the language system. While the overlap of the covered anatomical space by ConProb and GS was generally high for both AF and IFOF, connections along the ILF found in the majority by SP and TD were rated as less likely by ConProb.

Regarding the strong concept of Gibbs tracking, the results for the dorsal pathway strongly correlated with the results of the other methods. This is in line with a successful application of this method on different tract systems of the brain [22]. The reason for only one successful reconstruction via the IFOF and only some reconstructions via the ILF could be the limited number of gradient directions. For a higher number, the method has shown excellent performance previously [22].

Regarding the global search, SP and TD, slightly better average FA values were observed for GS than for SP or TD, which might be due to the inclusion of FA into the cost function. As a result, the search space is preferably expanded to regions with higher anisotropic diffusion, which may result in fibers located closer towards the center of pathways.

While GS was able to reconstruct the AF and in particular the IFOF with initial settings in most cases, both SP and TD required a reduction of the FA threshold and padding of the ROI volume in order to establish a connection at all. Moreover, visual inspection of fiber tracts resulting from reconstruction with SP and TD showed a tendency to erroneously reconstruct fibers along the ILF rather than along the IFOF. The difficulties of both deterministic approaches to reconstruct the AF, and the IFOF in particular, can be attributed to the design of the algorithms, which only take the local tensor information into account for fiber reconstruction. Especially for pathways with multiple terminations such as the IFOF [54], this local interpretation of the tensor data is not sufficient, which makes deterministic approaches susceptible to tracking errors.

On the other hand, global approaches like GS make use of the entire tensor information and establish connections which are optimal in respect of the available diffusion data between two ROIs. The underlying concept of cost minimization is justified since it is anticipated that functional areas are connected by preferably short and direct pathways as a result of the morphological evolution of white matter pathways in the brain [55]. Additionally, since multiple potential fiber directions are considered simultaneously, GS appropriately handles uncertainty in case of splitting and merging fibers. In this sense, GS [25] is similar to global approaches like probabilistic fiber tracking [19] or the shortest path approach using a Bayesian framework [24], which all implicitly model the uncertainty of DTI data.

However, since GS avoids time-consuming computations, it can be considered as a compromise between these approaches and deterministic fiber tracking. As anatomical connectivity can be resolved with sufficient accuracy and results are provided in a fast way, GS is well suited for clinical application. In particular, in combination with DTI protocols that acquire smaller numbers of gradient images and with clearly defined start and end ROIs located by fMRI [10], GS can provide information about the location of neuronal pathways for both pre-operative planning and intra-operative neurosurgery. Moreover, since the approach itself is independent of the underlying diffusion model, its extension to more complex diffusion models based on high angular resolution diffusion imaging such as q-ball imaging [56] or higher order tensors [57] is straight forward.

Despite of its considerable advantages, one limitation of the GS approach remains. In case of functional areas that are connected via multiple alternative pathways, GS requires anatomical knowledge to appropriately define the search space with the bounding box. However, as this mechanism can be used to draw the focus to either pathway, GS is the superioralternative to investigate anatomical connectivity, since SP and TD are not able to take advantage of such an approach.

## Conclusion

In this study, two deterministic approaches to fiber tracking, streamline propagation (SP) and tensor deflection (TD), a dedicated approach for anatomical connectivity analysis (GS), Gibbs tracking (GibbsT) and a connection-oriented probabilistic tractography approach (ConProb) were compared with respect to their capability to reconstruct neuronal pathways of the language system. Based on DTI data of a patient group with supratentorial tumors close to pathways and functional areas, and of a control group, two pathways of the semantic system were considered. In addition to visual validation based on anatomical knowledge, the evaluation of diffusion values and distances between fiber tracts showed that GS produces superior results as compared to SP, TD, and GibbsT. In particular, the ventral language connections via the IFOF were revealed by GS in most cases which was in line with the probability maps of ConProb. In contrast, SP, TD, and GibbsT often failed with standard settings for ROIs and thresholds on fractional anisotropy (FA). Despite relaxation of FA thresholds and expansion of ROIs, SP, TD, and GibbsT still failed in some cases. Overall, GS proved to be more reliable with less failures and less adjustments required.

## References

[1] Duffau H, Gatignol P, Mandonnet E, Peruzzi P, Tzourio-Mazoyer N, et al (2005) New insights into the anatomo-functional connectivity of the semantic system: A study using cortico-subcortical electrostimulations. Brain 128: 797–810. 15705610 10.1093/brain/awh423

[2] Goebel R, Esposito F, Formisano E (2006) Analysis of functional image analysis contest (FIAC) data with BrainVoyager QX: From single-subject to cortically aligned group general linear model analysis and self-organizing group independent component analysis. Hum Brain Mapp 27: 392–401. 16596654 10.1002/hbm.20249 PMC6871277

[3] Saur D, Schelter B, Schnell S, Kratochvil D, Küpper H, et al (2010) Combining functional and anatomical connectivity reveals brain networks for auditory language comprehension. NeuroImage 49: 3187–3197. 19913624 10.1016/j.neuroimage.2009.11.009

[4] Friston K, Li B, Daunizeau J, Stephan K (2011) Network discovery with DCM. NeuroImage 56: 1202–1221. 21182971 10.1016/j.neuroimage.2010.12.039 PMC3094760

[5] Bello L, Gambini A, Castellano A, Carrabba G, Acerbi F, et al (2008) Motor and language DTI fiber tracking combined with intraoperative subcortical mapping for surgical removal of gliomas. NeuroImage 39: 369–382. 17911032 10.1016/j.neuroimage.2007.08.031

[6] Basser P, Pierpaoli C (1996) Microstructural and physiological features of tissues elucidated by quantitative diffusion tensor MRI. J Magn Reson B 111: 209–219. 8661285 10.1006/jmrb.1996.0086

[7] Nimsky C, Ganslandt O, Hastreiter P, Wang R, Benner T, et al (2007) Preoperative and intraoperative diffusion tensor imaging-based fiber tracking in glioma surgery. Neurosurgery 61: 178–186. 18813171 10.1227/01.neu.0000279214.00139.3b

[8] Golby AJ, Kindlmann G, Norton I, Yarmarkovich A, Pieper S, et al (2011) Interactive diffusion tensor tractography visualization for neurosurgical planning. Neurosurgery 68: 496–505. 21135713 10.1227/NEU.0b013e3182061ebb PMC3112275

[9] Kuhnt D, Bauer M, Becker A, Merhof D, Zolal A, et al (2012) Intraoperative visualization of _ber tracking based reconstruction of language pathways in glioma surgery. Neurosurgery 70: 911–920. 21946508 10.1227/NEU.0b013e318237a807

[10] Schonberg T, Pianka P, Hendler T, Pasternak O, Assaf Y (2006) Characterization of displaced white matter by brain tumors using combined DTI and fMRI. NeuroImage 30: 1100–1111. 16427322 10.1016/j.neuroimage.2005.11.015

[11] Nimsky C, Ganslandt O, Hastreiter P, Wang R, Benner T, et al (2005) Intraoperative diffusiontensor MR imaging: Shifting of white matter tracts during neurosurgical procedures–initial experience. Radiology 234: 218225. 10.1148/radiol.2341031984 15564394

[12] Maesawa S, Fujii M, Nakahara N, Watanabe T, Wakabayashi T, et al (2010) Intraoperative tractography and motor evoked potential (MEP) monitoring in surgery for gliomas around the corticospinal tract. World Neurosurgery 74: 153–161. 21300007 10.1016/j.wneu.2010.03.022

[13] Basser PJ, Pajevic S, Pierpaoli C, Duda J, Aldroubi A (2000) In vivo fiber tractography using DT-MRI data. Magn Reson Med 44: 625–632. 11025519 10.1002/1522-2594(200010)44:4<625::aid-mrm17>3.0.co;2-o

[14] Lazar M, Alexander A (2003) An error analysis of white matter tractography methods: Synthetic diffusion tensor field simulations. NeuroImage 20: 1140–1153. 14568483 10.1016/S1053-8119(03)00277-5

[15] Mori S, Crain B, Chacko V, van Zijl P (1999) Three-dimensional tracking of axonal projections in the brain by magnetic resonance imaging. Ann Neurol 45: 265–269. 9989633 10.1002/1531-8249(199902)45:2<265::aid-ana21>3.0.co;2-3

[16] Parker G, Wheeler-Kingshott C, Barker G (2002) Estimating distributed anatomical connectivity using fast marching methods and diffusion tensor imaging. Proc IEEE Trans Med Imaging 21: 505–512. 10.1109/TMI.2002.1009386 12071621

[17] Jackowski M, Kao C, Qiu M, Constable R, Staib L (2005) White matter tractography by anisotropic wavefront evolution and diffusion tensor imaging. Med Image Anal 9: 427–440. 16040268 10.1016/j.media.2005.05.008 PMC2839167

[18] Parker G, Haroon H, Wheeler-Kingshott C (2003) A framework for a streamline-based probabilistic index of connectivity (PICo) using a structural interpretation of MRI diffusion measurements. J Magn Reson Imaging 18: 242–254. 12884338 10.1002/jmri.10350

[19] Kreher B, Schnell S, Mader I, Il'yasov KA, Hennig J, et al (2008) Connecting and merging fibres: Pathway extraction by combining probability maps. NeuroImage 43: 81–89. 18644243 10.1016/j.neuroimage.2008.06.023

[20] Reisert M, Mader I, Kiselev V (2009) Global reconstruction of neuronal fibers. In: Proc. MICCAI Diffusion Modelling Workshop.

[21] Fillard P, Descoteaux M, Goh A, Gouttard S, Jeurissen B, et al (2011) Quantitative evaluation of 10 tractography algorithms on a realistic diffusion MR phantom. NeuroImage 56: 220–234. 21256221 10.1016/j.neuroimage.2011.01.032

[22] Reisert M, Mader I, Anastasopoulos C, Weigel M, Schnell S, et al (2011) Global fiber reconstruction becomes practical. NeuroImage 54: 955–962. 20854913 10.1016/j.neuroimage.2010.09.016

[23] Cheng P, Magnotta V, Wu D, Nopoulos P, Moser D, et al (2006) Evaluation of the GTRACT diffusion tensor tractography algorithm: A validation and reliability study. NeuroImage 31: 1075–1085. 16631385 10.1016/j.neuroimage.2006.01.028

[24] Zalesky A (2008) DT-MRI fiber tracking: A shortest paths approach. In: Proc. IEEE Trans Med Imaging. volume 27, pp. 1458–1471. 10.1109/TMI.2008.923644 18815098

[25] Merhof D, Richter M, Enders F, Hastreiter P, Ganslandt O, et al.. (2006) Fast and accurate connectivity analysis between functional regions based on DT-MRI. In: Proc. MICCAI. pp. 225–233 (Part II). 10.1007/11866763_28 17354776

[26] Dechter R, Pearl J (1985) Generalized best-first search strategies and the optimality of A*. J ACM 32: 505–536.

[27] Parker G, Luzzi S, Alexander D, Wheeler-Kingshott C, Ciccarelli O, et al (2005) Lateralization of ventral and dorsal auditory-language pathways in the human brain. NeuroImage 24: 656–666. 15652301 10.1016/j.neuroimage.2004.08.047

[28] Duffau H (2008) The anatomo-functional connectivity of language revisited. New insights provided by electrostimulation and tractography. Neuropsychologia 46: 927–934. 18093622 10.1016/j.neuropsychologia.2007.10.025

[29] Turken A, Dronkers N (2011) The neural architecture of the language comprehension network: Converging evidence from lesion and connectivity analyses. Front Syst Neurosci 5. 10.3389/fnsys.2011.00001 PMC3039157 21347218

[30] Mandonnet E, Nouet A, Gatignol P, Capelle L, Duffau H (2007) Does the left inferior longitudinal fasciculus play a role in language? A brain stimulation study. Brain 130: 623–629. 17264096 10.1093/brain/awl361

[31] Kier E, Staib L, Davis L, Bronen R (2004) MR imaging of the temporal stem: Anatomic dissection tractography of the uncinate fasciculus, inferior occipitofrontal fasciculus, and Meyers loop of the optic radiation. American Journal of Neuroradiology 25: 677–691. 15140705 PMC7974480

[32] Martino J, Brogna C, Robles S, Vergani F, Duffau H (2010) Anatomic dissection of the inferior fronto-occipital fasciculus revisited in the lights of brain stimulation data. Cortex 46: 691–699. 19775684 10.1016/j.cortex.2009.07.015

[33] Bertani G, Carrabba G, Raneri F, Fava E, Castellano A, et al (2012) Predictive value of inferior fronto-occipital fasciculus (IFO) DTI-fiber tracking for determining the extent of resection for surgery of frontal and temporal gliomas preoperatively. J Neurosurg Sci 56: 137–143. 22617176

[34] Mohades S, Struys E, Schuerbeek PV, Mondt K, Craen PVD, et al (2012) DTI reveals structural differences in white matter tracts between bilingual and monolingual children. Brain Research 1435: 137–143. 10.1016/j.brainres.2011.12.005 22197702

[35] McDonald C, Ahmadi M, Hagler D, Tecoma E, Iragui V, et al (2008) Diffusion tensor imaging correlates of memory and language impairments in temporal lobe epilepsy. Neurology 71: 1869–1876. 18946001 10.1212/01.wnl.0000327824.05348.3b PMC2676974

[36] Vandermosten M, Boets B, Poelmans H, Sunaert S, Wouters J, et al (2012) A tractography study in dyslexia: Neuroanatomic correlates of orthographic, phonological and speech processing. Brain 135: 935–948. 22327793 10.1093/brain/awr363

[37] Schmahmann J, Pandya D (2006) Fiber Pathways of the Brain, Oxford University Press, chapter 18,19.

[38] Mann HB, Whitney DR (1947) On a test of whether one of two random variables is stochastically larger than the other. Annals of Mathematical Statistics 18: 50–60.

[39] Thesen S, Heid O, Mueller E, Schad L (2000) Prospective acquisition correction for head motion with image-based tracking for real-time fMRI. Magn Reson Med 44: 457–465. 10975899 10.1002/1522-2594(200009)44:3<457::aid-mrm17>3.0.co;2-r

[40] Basser P, Pierpaoli C (1998) A simplified method to measure the diffusion tensor from seven MR images. Magn Reson Med 39: 928–934. 9621916 10.1002/mrm.1910390610

[41] Hasan K, Parker D, Alexander A (2001) Comparison of gradient encoding schemes for diffusiontensor MRI. J Magn Reson Imaging 13: 769–780. 11329200 10.1002/jmri.1107

[42] Lebel C, Benner T, Beaulieu C (2011) Six is enough? comparison of diffusion parameters measured using six or more diffusion-encoding gradient directions with deterministic tractography. Magn Reson Med 68: 474–483. 22162075 10.1002/mrm.23254

[43] Jones D (2008). PISTE - Phantom images for simulating tractography errors. http://cubric.psych.cf.ac.uk/commondti. Cardiffniversity Brain Research Imaging Centre (CUBRIC), [Online; accessed 20-June-2012].

[44] Laganà M, Rovaris M, Ceccarelli A, Venturelli C, Marini S, et al (2010) DTI parameter optimisation for acquisition at 1.5T: SNR analysis and clinical application. Computational Intelligence and Neuroscience 2010: 8 pages. 10.1155/2010/254032 PMC2804108 20069121

[45] Westin CF, Peled S, Gubbjartsson H, Kikinis R, Jolesz F (1997) Geometrical diffion measures for MRI from tensor basis analysis. In: ISMRM '97. p. 1742.

[46] Voineskos A, Lobaugh N, Bouix S, Rajji T, Miranda D, et al (2010) Diffion tensor tractography fiings in schizophrenia across the adult lifespan. Brain 133: 1494–504. 20237131 10.1093/brain/awq040 PMC2859148

[47] Ding Z, Gore J, Anderson A (2001) Case study: Reconstruction, visualization and quantification of neuronal fiber pathways. In: Proc. IEEE Visualization. pp. 453–456.

[48] Zhang S, Laidlaw DH (2002) Hierarchical clustering of streamtubes. Technical Report CS-02–18.

[49] Brun A, Park HJ, Knutsson H, Westin CF (2003) Coloring of DT-MRI fir traces using Laplacian eigenmaps. In: Computer Aided Systems Theory (EUROCAST'03), Lecture Notes in Computer Science 2809. pp. 564–572.

[50] Corouge I, Gouttard S, Gerig G (2004) Towards a shape model of white matter fir bundles using di_usion tensor MRI. In: Proc. IEEE Int Symp Biomed Imaging. volume 1, pp. 344–347.

[51] Neher P, Stieltjes B, Reisert M, Reicht I, Meinzer HP, et al (2012) MITK global tractography. Proceedings of the SPIE, Medical Imaging 8314: 83144D–6.

[52] Aganj I, Lenglet C, Sapiro G (2009) ODF reconstruction in q-ball imaging with solid angle consideration. In: Proceedings of the Sixth IEEE international conference on Symposium on Biomedical Imaging: From Nano to Macro. ISBI'09, pp. 1398–1401. 10.1109/ISBI.2009.5193327 PMC4360965 25789084

[53] Kreher B, Hennig J, Il'yasov K (2006) DTI & FiberTools: A complete toolbox for DTI calculation, fiber tracking, and combined evaluation. In: Proceeding of ISMRM 14th International Scientific Meeting.

[54] Martino J, Brogna C, Robles S, Vergani F, Duff H (2010) Anatomic dissection of the inferior fronto-occipital fasciculus revisited in the lights of brain stimulation data. Cortex 46: 691–699. 19775684 10.1016/j.cortex.2009.07.015

[55] van Essen D (1997) A tension-based theory of morphogenesis and compact wiring in the central nervous system. Nature 385: 313–318. 9002514 10.1038/385313a0

[56] Tuch D (2004) Q-ball imaging. Magn Reson Med 52: 1358–1372. 15562495 10.1002/mrm.20279

[57] Oezarslan E, Mareci T (2003) Generalized diffsion tensor imaging and analytical relationships between diffion tensor imaging and high angular resolution diffion imaging. Magn Reson Med 50: 955–965. 14587006 10.1002/mrm.10596

